# Structural Study of a New MbtI-Inhibitor Complex: Towards an Optimized Model for Structure-Based Drug Discovery

**DOI:** 10.3390/ph16111559

**Published:** 2023-11-03

**Authors:** Matteo Mori, Stefania Villa, Laurent R. Chiarelli, Fiorella Meneghetti, Marco Bellinzoni

**Affiliations:** 1 Department of Pharmaceutical Sciences, University of Milan, Via L. Mangiagalli 25, 20133 Milano, Italy; matteo.mori@unimi.it (M.M.); stefania.villa@unimi.it (S.V.); fiorella.meneghetti@unimi.it (F.M.); 2 Department of Biology and Biotechnology “Lazzaro Spallanzani”, University of Pavia, Via A. Ferrata 9, 27100 Pavia, Italy; laurent.chiarelli@unipv.it; 3 Institut Pasteur, Université Paris Cité, CNRS UMR3528, Unité de Microbiologie Structurale, F-75015 Paris, France

**Keywords:** *Mycobacterium tuberculosis*, salicylate synthase, siderophore, iron, co-crystal structure, anti-virulence therapy, tuberculosis

## Abstract

MbtI from *Mycobacterium tuberculosis* (*Mtb*) is a Mg^2+^-dependent salicylate synthase, belonging to the chorismate-utilizing enzyme (CUE) family. As a fundamental player in iron acquisition, MbtI promotes the survival and pathogenicity of *Mtb* in the infected host. Hence, it has emerged in the last decade as an innovative, potential target for the anti-virulence therapy of tuberculosis. In this context, 5-phenylfuran-2-carboxylic acids have been identified as potent MbtI inhibitors. The first co-crystal structure of MbtI in complex with a member of this class was described in 2020, showing the enzyme adopting an open configuration. Due to the high mobility of the loop adjacent to the binding pocket, large portions of the amino acid chain were not defined in the electron density map, hindering computational efforts aimed at structure-driven ligand optimization. Herein, we report a new, high-resolution co-crystal structure of MbtI with a furan-based derivative, in which the closed configuration of the enzyme allowed tracing the entirety of the active site pocket in the presence of the bound inhibitor. Moreover, we describe a new crystal structure of MbtI in open conformation and in complex with the known inhibitor methyl-AMT, suggesting that in vitro potency is not related to the observed enzyme conformation. These findings will prove fundamental to enhance the potency of this series via rational structure-based drug-design approaches.

## 1. Introduction

Iron is a fundamental cofactor in most bacterial species, including mycobacteria [1,2,3]. In mammals, it is mainly bound in its oxidized form (Fe^3+^) to iron-storage or transport proteins, due to its toxicity and low solubility [4,5]. As pathogenic microorganisms need this essential metal to ensure their survival and sustain their pathogenicity, most higher organisms, including humans, have evolved an innate defensive strategy based on the restriction of the amount of available extracellular iron [6,7,8,9]. Nevertheless, many pathogens, mainly bacteria and fungi, have developed efficient countermeasures to compensate for this effect [10,11,12]. In mycobacteria, one of the most important iron acquisition pathways relies on the production of siderophores [3,6,13]. These low-molecular-weight chelators, known as carboxymycobactins (cMBTs) and mycobactins (MBTs), share a common core but differ in their terminal portion, leading to different solubility and functional properties. While the hydrophilic cMBTs are released in the extracellular environment, where they actively scavenge for iron, the lipophilic MBTs remain anchored to the mycobacterial wall, receiving iron from cMBTs and shuttling it towards the cytoplasm. These mechanisms are exhaustively described in a number of review articles [3,6,14,15].

The targeting of siderophores, and, more generally, metallophores, has seen increasing interest as a viable opportunity for the anti-virulence therapy of a variety of infections [16,17]. Anti-virulence compounds represent the new frontier of antimicrobial treatment; they act on non-essential enzymes that are, however, required when the pathogen infects the host [18,19,20]. As a result, the therapeutic agent does not exert a constant, selective pressure on the microorganism, thus contributing to preventing resistance phenomena (multi-drug resistance or MDR and extensive drug resistance or XDR), a major issue for the treatment of tuberculosis [21,22,23].

The first enzyme involved in the production of siderophores in *Mycobacterium tuberculosis* (*Mtb*) is MbtI, a member of the chorismate-utilizing enzyme (CUE) family and the menaquinone, siderophore, and tryptophan (MST) subfamily [24,25]. MbtI converts chorismate to salicylate via a two-step mechanism, with the release of pyruvate [26,27]. Salicylate then becomes the building block for the core scaffold of all siderophores, which are assembled by a large complex, comprising three non-ribosomal peptide synthetases (NRPS, MbtB, MbtE, MbtF) and two polyketide synthases (MbtC, MbtD), upon activation of the acidic moiety by MbtA [3]. Additionally, MbtG catalyzes the *N*-hydroxylation of the l-Lys portion, and MbtH assists the folding of the NRPSs; the function of MbtJ is still unknown. The formation of the terminal chain is mediated by MbtL, MbtM, and MbtN, while its fusion to the core scaffold is catalyzed by MbtK [3]. Because they act upstream in the biosynthetic pathway, MbtI and MbtA have been widely investigated as potential targets for anti-virulence therapeutic strategies against tuberculosis (TB) [6,28,29,30]. Studies on these two enzymes have led to numerous potent inhibitors, with promising antimycobacterial activities both on isolated strains and infected macrophage models [31,32,33,34,35,36,37,38,39,40,41,42].

**Figure 1:**
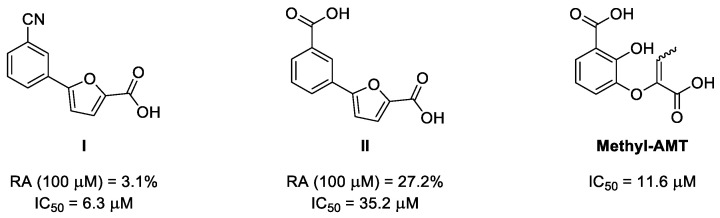
Chemical structure and activity of the two furan-based inhibitors **I** and **II** [35] and methyl-AMT [31,38], which were co-crystallized with MbtI. Biological effects are expressed as % of residual enzymatic activity (RA) and half maximal inhibitory concentration (IC_50_).

The crystal structure of MbtI was first reported in 2006 by Harrison and co-workers [26]. Since then, many studies have been dedicated to the structural analysis of this enzyme, with a particular emphasis on the well-known plasticity of its active site; several co-crystal structures have also been investigated, mainly with derivatives of the inhibitor 3-(1-carboxyprop-1-enyloxy)-2-hydroxybenzoic acid (methyl-AMT) [38,43]. In 2020, the first complex with a 5-phenylfuran-2-carboxylic acid analog (**I**, Figure 1) was described [35]. This class of compounds has emerged as the most promising on MbtI and has also shown significant antibacterial effect on *Mtb* [37]. Therefore, the study of the binding of these compounds is of particular interest for their optimization. MbtI is known to exist in two possible conformations, generally referred to as “open” and “closed”, depending on the position of two flexible loops relative to the active site [26], as extensively analyzed in our previous work [35]. The careful inspection of the structure provided crucial information on the inhibition mechanism and suggested that the enzyme conformation in the crystal structure did not correlate with the biological activity of the inhibitor. This conclusion was consistent with previous work by Manos-Turvey and co-workers, who observed a closed configuration of the enzyme for the least active derivative and open forms for more interesting candidates [44]. Similarly, the most potent member of our series was co-crystallized with MbtI into an open state; as a result, there was no supporting electron density for the flexible loops covering the active site pocket in the closed state [35]. This lack of definition in areas adjacent to the active site complicated further optimization efforts by means of computational studies, because of the inherent difficulty of modeling relatively large and mobile portions of the enzyme.

In this work, we describe the co-crystal structure of MbtI with 5-(3-carboxyphenyl)furan-2-carboxylic acid (**II** [35], Figure 1) in a closed conformation, as well as a new complex with the known inhibitor methyl-AMT, characterized by an open conformation, which contrasts with the previously published structure (PDB code: 3VEH) [43]. The high resolution of the binding cavity, shaped around the furan-based ligand, will provide new, key information for the rational design of optimized inhibitors. Our efforts will hopefully contribute on multiple levels to the advancement of the current therapeutic approaches against TB.

## 2. Results and Discussion

**Figure 2:**
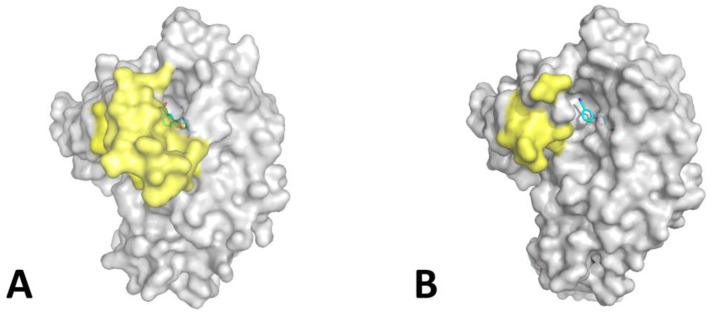
Comparison between the MbtI-**II** (**A**) and MbtI-**I** (**B**) structures. In yellow are shown the mobile loops (only partially traced in **B** due to the lack of supporting electron density in some regions). Bound ligands are shown as sticks, with carbon atoms in cyan.

In the context of our efforts to elucidate the binding mode of 5-phenylfuran-2-carboxylic acids to MbtI [35], we obtained co-crystals with 5-(3-carboxyphenyl)furan-2-carboxylic acid (**II**). Despite its modest inhibitory activity in vitro (IC50: 35.2 ± 2.6 μM; residual activity at 100 μM: 27.2% ± 4.5) [35], the compound was chosen as part of a large panel of crystallization trials. As rod-shaped crystals were obtained after a few weeks at 4 °C, we welcomed the opportunity to examine a new MbtI-inhibitor complex, not only to characterize the ligand pose but also to study the conformation of the loops in the proximity of the binding pocket. MbtI-**II** crystals are not isomorphous to those obtained for the MbtI-**I** complex (PDB code: 6ZA4) [35], nor to other MbtI crystal structures currently available in the PDB, and the analysis of the resulting model revealed the presence of two molecules in the asymmetric unit (ASU). However, the major discrepancy when compared to the MbtI-**I** complex was the position of the flexible loops (residues 268−293 and 324−336) with respect to the active site (Figure 2).

**Figure 3:**
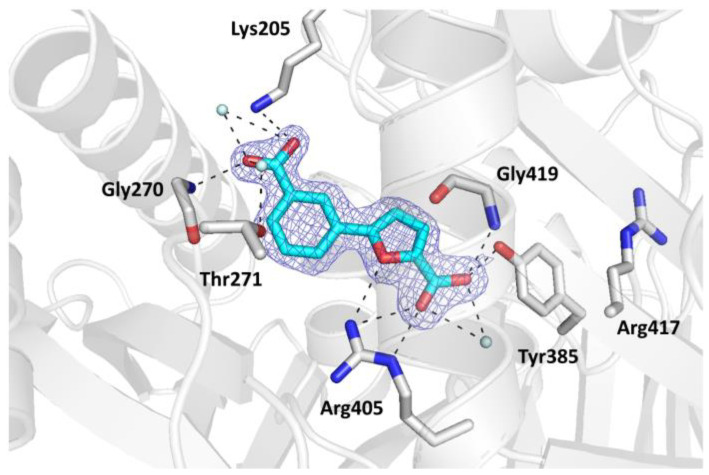
Ribbon diagram of the MbtI-**II** co-crystal structure: the interactions of **II** with the amino acid residues (in sticks) and the water molecules (cyan spheres) are represented as dashed lines. The blue mesh represents the electron density around the ligand (contoured at 1σ).

Interestingly, MbtI was found to be in a closed conformation, thus reproducing the arrangement of the loops found by Manos-Turvey et al. in an apo form of the protein (PDB code: 3LOG), and by our group in the complex with the natural product salicylate and Mg^2+^ (PDB code: 6ZA5) [35,38]. The rms distances between the Ca carbons of residues 325−335 for MbtI-**II**/3LOG and MbtI-**II**/6ZA5 were measured to be in the range 0.19−0.46 Å and 0.34−0.56 Å, respectively, showing a considerable similarity between the overall enzyme conformation among the MbtI protomers in the three complexes. In the case of MbtI-**II**, loop stabilization may have contributed to improve the overall resolution of the crystallographic data (up to 1.6 Å), leading to a significantly higher value than the one obtained for the MbtI-**I** dataset. Moreover, the electron density map for the active site region was clearly interpretable in all its parts (Figure 3), effectively providing a complete picture of the active site region around the ligand. In our previous paper, we proposed that the closed conformation may be stabilized by the formation of H-bonds between the ligand and Gly270 and/or Thr271, causing the first mobile loop (and, in turn, the second loop) to be dragged towards the active site [35]. In agreement with this hypothesis, the analysis of the ligand pose revealed the presence of an H-bond between the carboxylic group at position 3 of the phenyl and the main chain amide of Gly270 (Figure 3).

**Figure 4:**
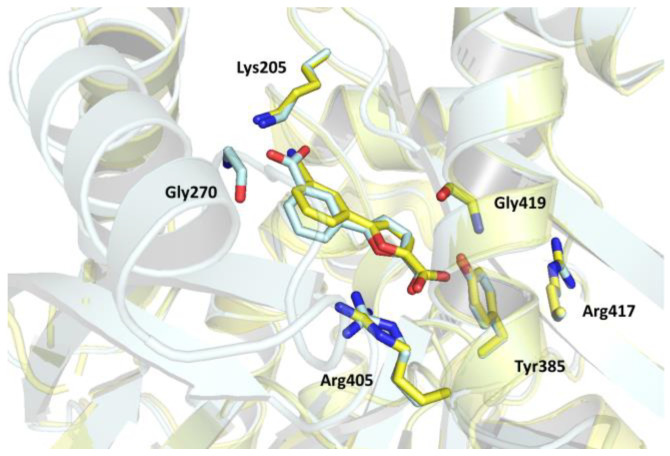
Superposition of the MbtI-**II** (cyan) and MbtI-**I** (yellow) structures, evidencing the similar binding orientation of the inhibitors.

The binding mode of the compound was equivalent in the two MbtI protomers. The carboxylic moiety of the furan established H-bonds with the side chains of Tyr385, Arg405, and with the main chain amide of Gly419. Moreover, it coordinated a water molecule, which was further bound to Arg405 and Arg417. The furan oxygen interacted with the guanidine group of Arg405. Finally, the carboxylic substituent on the phenyl ring formed H-bonds with Gly270 (as previously mentioned) and two ordered water molecules; one of them established a further interaction with the hydroxyl group of Thr271, while the other did so with the amine of Lys205. These two contacts are significant, both from a structural and functional point of view. The former further reinforced the stabilization of the mobile loops, adding up to the Gly270 interaction. The observed interaction with Lys205, one of the key catalytic residues, is noteworthy and may contribute to explain the lower inhibitor potency. In our previous work, we speculated that the superior activity of **I** could be due to its ability to strongly bind this amino acid through its cyano group. This hypothesis is consistent with the present structure, which suggests a weaker interaction between compound **II** and MbtI Lys205, as evinced by the presence of an H-bond interaction between the ligand carboxylic group and the lysine ε-amino group, but a higher C-N distance (~3.20 Å) than that observed in the MbtI-**I** complex (2.29–2.82 Å over the four chains of the ASU). The reason for this discrepancy does not seem to reside in a different orientation of the side chain of Lys205 in the present complex, but rather in the length and geometry of the compound **I** cyano moiety, which is able to reach the Lys205 ε-amino group more closely than the carboxylic substituent of compound **II**. Otherwise, the comparison of the two MbtI complexes revealed a substantial preservation of the binding pose of either compound (Figure 4), with a remarkable superimposition of the 2-furoic acid moiety, involved in the same interactions with the enzyme in both complexes. Conversely, the phenyl group was relatively more unconstrained and thus freer to assume slightly different arrangements. In any case, the near-perfect overlap of the two ligands, although expected, is noteworthy and implies that ligand binding is not significantly influenced by the MbtI overall conformation. On the other hand, it supports the hypothesis that the induced closure of the active site pocket does not directly correlate with the biological activity of the compound and that other, more consequential factors are at play when it comes to the inhibitory effect.

**Figure 5:**
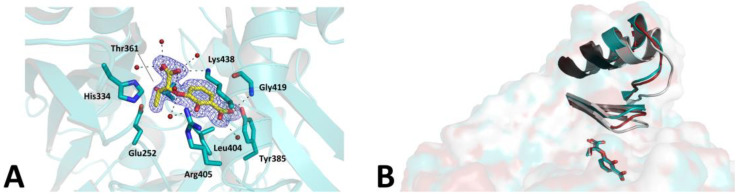
(**A**). Ribbon diagram of the MbtI-methyl-AMT co-crystal structure: the interactions of the compound (yellow sticks) with surrounding residues (teal sticks) and water molecules (red spheres) are represented as dashed lines. The blue mesh represents the electron density around the ligand (contoured at 1σ). (**B**). Superposition of MbtI-methyl-AMT_B (teal; this work), 3VEH_B (silver), and 3VEH_D (red). The mobile loops (residues 273–284 and 327–331) are shown as ribbons, while the ligands are represented as sticks.

This point is further supported by a novel complex, solved at 1.5 Å resolution, between MbtI and the inhibitor methyl-AMT (*Z* isomer), which shows, in comparison with the previously reported structure [43], an equivalent inhibitor pose (Figure 5A) but a different enzyme conformation (Figure 5B). In the published model (PDB code: 3VEH), three out of four chains in the ASU (A-C) showed mobile loops closed upon the active site in a sort of lid, while in the fourth chain (chain D in PDB 3VEH) they were tilted upwards, de facto into an open conformation. Interestingly, in our MbtI-methyl-AMT model, all protomers were rather found to be in an open state, with poor or no supporting electron density for most of the mobile loops (residues 273–284 and 327–331) in three out of the four protomers in the ASU. However, these regions could be traced entirely in the fourth protomer (chain B), mostly due to stabilizing packing interactions involving the Gly277-Ile280 β-turn. As a result, the conformation of this MbtI protomer reproduced the arrangement observed for chain D in the previously published model (3VEH), the two chains superimposing with an RMSD of 0.54 Å (Figure 5B). However, contrarily to the previous complex, and despite an equivalent pose in the active site, no hydrogen bond interaction could be observed in our complex between the methyl-AMT enolpyruvyl side chain and the hydroxyl group of Thr271, supporting the hypothesis of a correlation between this interaction and the closed state of the enzyme. These findings are in line with our conclusion that the overall MbtI conformation, and most notably the closure of the active site lid, does not correlate with the inhibitor potency in vitro, with different protomers showing different conformations in the presence of the same compound.

Overall, our data indicated that members of the 5-phenylfuran-2-carboxylic acid class, which show varying in vitro potencies, interact with MbtI with the same binding mode, irrespective of the conformation of the enzyme. This finding is noteworthy from a drug-design standpoint because it provides a new framework for structure-based compound optimization. Therefore, considering the ability of the active site to undergo significant structural rearrangements around substrates and inhibitors, the design of the new derivatives would greatly benefit from the availability of experimental structures featuring MbtI in two different conformations. In addition, this information will provide useful insights for the development of a computational model for virtual screening approaches. Most notably, we expect that this new tool will aid the design of inhibitors capable of being accommodated into the binding pocket while exploiting new contacts with the ensemble of the active site environment. Moreover, considering the intrinsic difficulties in obtaining compounds active on *M. tuberculosis*, drug-design efforts will also have to consider strategies to increase the lipophilicity of the compounds to enhance their penetration of the mycolic acid-rich pathogen cell wall. Therefore, the new structural data will guide the insertion of suitable moieties, without impacting on the enzymatic activity of the compounds. Finally, considering the significant evolutionary conservation of MST enzymes within the *Mycobacterium* genus, our findings may be also applied to homologs from different microorganisms for which experimental structural data are still unavailable [45].

## 3. Materials and Methods

### 3.1. Chemistry

All starting materials, chemicals, and solvents were purchased from commercial suppliers (Sigma-Aldrich, St. Louis, MO, USA; FluoroChem, Hadfield, UK) and used as received. Anhydrous solvents were utilized without further drying. The course of the reactions was monitored by thin-layer chromatography (TLC), using aluminum-backed silica gel 60 plates (0.2 mm; Merck, Darmstadt, Germany). Microwave-assisted reactions were carried out with a Biotage^®^ Initiator Classic (Biotage, Uppsala, Sweden). Silica gel 60 (40−63 μm; Merck) was used for the purification of the compounds by flash column chromatography. The identity of the molecules was verified by NMR spectroscopy on a Varian Oxford instrument (Varian, Palo Alto, CA, USA), operating at 300 MHz and 75 MHz for ^1^H and ^13^C, respectively. Melting points were determined in open capillary tubes on a Stuart SMP30 melting point apparatus (Cole-Parmer Stuart, Stone, UK). Analytical data are in accordance with previously published data.

**Scheme 1:**
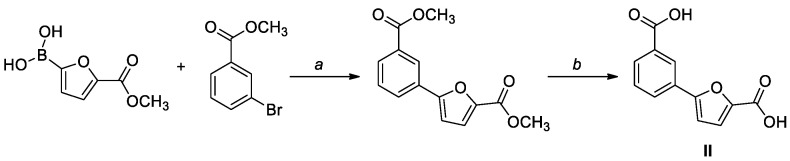
Main reactions leading to **II**. Reagents and conditions: (*a*) Pd(PPh_3_)_2_Cl_2_, 2 M Na_2_CO_3_, 1,4-dioxane, 60 °C, 90 min, MW, N_2_; (b) NaOH, EtOH/THF 1:1, reflux, 5 h.

5-(3-Carboxyphenyl)furan-2-carboxylic acid (**II**) was obtained as previously described [35]. Briefly, (5-(methoxycarbonyl)furan-2-yl)boronic acid was reacted with methyl 3-bromobenzoate in a Suzuki−Miyaura coupling; the resulting intermediate was hydrolyzed in basic conditions to afford the desired diacid **II** (Scheme 1). The synthesis of methyl-AMT followed the procedure published by Manos-Turvey and co-workers [38], and reproduced by our group in a previous paper [31]. All spectral data were consistent with the expected values [31,35].

5-(3-Carboxyphenyl)furan-2-carboxylic acid (**II**). Aspect: white solid. mp > 300 °C. ^1^H NMR (300 MHz, DMSO-*d*_6_): δ (ppm) 13.20 (bs exch. D_2_O, 2H, COOH), 8.30 (s, 1H, H_Ar_), 8.04 (d, *J* = 7.8, 1H, H_Ar_), 7.92 (d, *J* = 7.8, 1H, H_Ar_), 7.60 (t, *J* = 7.8 Hz, 1H, H_Ar_), 7.32 (d, *J* = 3.6 Hz, 1H, H_Het_), 7.25 (d, *J* = 3.6 Hz, 1H, H_Het_). ^13^C NMR (75 MHz, DMSO-*d*_6_): δ (ppm) 167.3 (COOH), 159.7 (COOH), 155.6 (C_Het_), 145.0 (C_Het_), 132.2 (C_Ar_), 130.0 (C_Ar_), 129.9 (C_Ar_), 129.1 (C_Ar_), 125.2 (C_Ar_), 120.2 (C_Het_), 109.2 (C_Het_).

3-(1-Carboxyprop-1-enyloxy)-2-hydroxybenzoic acid (Me-AMT). *Z*/*E* ratio: 7.4/2.6. Aspect: off-white solid. mp: 207–209 °C (dec.). ^1^H NMR (300 MHz, DMSO-*d_6_*/D_2_O) δ (ppm) 7.45 (dd, *J* = 8.0, 1.6 Hz, 1H, *E*-H_Ar_), 7.40 (dd, *J* = 8.0, 1.6 Hz, 1H, *Z*-H_Ar_), 7.00 (dd, *J* = 8.0, 1.6 Hz, 1H, *E*-H_Ar_), 6.85 (dd, *J* = 8.0, 1.6 Hz, 1H, *Z*-H_Ar_), 6.80–6.69 (m, 2H, *E*-H_Ar_ + *Z*-H_Ar_), 6.61 (q, *J* = 7.1 Hz, 1H, *Z*-CH), 5.80 (q, *J* = 7.5 Hz, 1H, *E*-CH), 1.96 (d, *J* = 7.5 Hz, 3H, *E*-CH_3_), 1.67 (d, *J* = 7.1 Hz, 3H, *Z*-CH_3_). ^13^C NMR (75 MHz, DMSO-*d_6_*/D_2_O) δ (ppm) 172.49 (*Z*-COOH), 172.24 (*E*-COOH), 164.35 (*E*-COOH), 163.66 (*Z*-COOH), 152.96 (*E*-C_Ar_), 151.70 (*Z*-C_Ar_), 146.06 (*Z*-C_Ar_), 145.97 (*E*-C_Ar_), 143.31 (*E*-C_Alk_), 142.45 (*Z*-C_Alk_), 126.67 (*Z*-C_Ar_), 124.34 (*E*-C_Ar_), 123.54 (*E*-C_Ar_), 123.23 (*Z*-C_Ar_), 122.10 (*E*-C_Ar_), 118.86 (*Z*-C_Ar_), 118.36 (*Z*-C_Ar_), 118.16 (*E*-C_Ar_), 115.49 (*E*-C_Alk_), 115.01 (*Z*-C_Alk_), 13.09 (*E*-CH_3_), 11.67 (*Z*-CH_3_).

### 3.2. Production and Purification of MbtI

The production and purification of MbtI was performed according to the previously published protocol [35]. In summary, *E. coli* BL21 (DE3) cells were transformed with a pET-28a plasmid, bearing a codon-optimized open reading frame for MbtI (GenScript, Piscataway, NJ, USA). After inducing expression by an autoinduction protocol, cultures were centrifuged, and the cell pellet was lysed with a CF2 cell disruptor (Constant Systems Ltd., Daventry, UK). The supernatant was charged on a HisTrap high-performance column (GE Healthcare, Chicago, IL, USA) and purified by affinity chromatography. Then, TEV protease was added to the protein solution to cleave the His-tag; the resulting mixture was dialyzed overnight at 4 °C. Subsequently, the solution was loaded on a Bio-Rad column (Bio-Rad, Hercules, CA, USA) charged with Ni-NTA resin to remove the His-tag. After a concentration step with Vivaspin 15R 10,000 Da MWCO centrifugal concentrators (Sartorius, Göttingen, Germany), the eluate was loaded on a HiLoad 16/600 Superdex 200 exclusion chromatography column (GE Healthcare), previously equilibrated in the final buffer (25 mM Hepes-NaOH pH 8.0, 150 mM NaCl, 1% glycerol). The collected fractions were concentrated to about 20 mg/mL, flash-frozen with liquid nitrogen, and stored at −80 °C.

### 3.3. Crystallization of MbtI Complexes

Crystallization experiments were performed at 4 °C by the sitting-drop vapor-diffusion technique in 96-well plates, according to established protocols at the Crystallography Core Facility of the Institut Pasteur (Paris, France) [46]. The trials were set up with a Mosquito crystal Nanoliter Protein Crystallization Robot (TTP Labtech, Melbourne, UK); the plates were stored in a Rock Imager 1000 (Formulatrix, Bedford, MA, USA) and visually checked by an online software. The drops were obtained by mixing an equal amount of protein and reservoir solutions to a final volume of 400 nL; the reservoir contained 150 μL of the precipitant mixture. MbtI, prepared as previously described, was incubated overnight at 4 °C with the ligands (**II** or methyl-AMT) at a final concentration of 5 mM. As for MbtI-**II**, rod-shaped crystals grew within 10–15 days in the presence of 15% PEG 8000 and 0.2 M (NH_4_)_2_SO_4_. Prismatic crystals of MbtI-methyl-AMT grew in a similar time frame in the presence of 16% PEG 3350, 0.1 M sodium citrate pH 5.6, and 2% tacsimate. The samples were harvested with CryoLoops (Hampton Research, Aliso Viejo, CA, USA), cryoprotected either in a 1:1 mixture of paraffin and Paratone-N oil (Hampton Research; MbtI-**II**) or in a mixture composed of 75% crystallization solution and 25% glycerol (MbtI-methyl-AMT), and flash-frozen in liquid nitrogen.

### 3.4. Data Collection and Analysis

Diffraction experiments were carried out at the SOLEIL Synchrotron facility (Saint-Aubin, France), on the PROXIMA-2A beamline. Data were processed, scaled, and analyzed using autoPROC [47]. The structure was solved by Molecular Replacement (MR) with PHASER [48], available in the CCP4 suite [49], using the published model of MbtI in complex with Ba^2+^ as the search model (PDB code: 6ZA6). Geometrical restraints for both inhibitor **II** and methyl-AMT were generated with the Grade server (http://grade.globalphasing.org (accessed on 5 September 2023)). Model rebuilding was performed by Coot [50], and the refinement was carried out with BUSTER (Version 2.10.3), applying local structure similarity restraints for non-crystallography symmetry [51] and a translation−libration−screw (TLS) model; TLS group definitions were optimized through the TLSMD server [52]. Models were validated with MolProbity [53] and Phenix [54]. Data collection, refinement, and statistics are summarized in Table 1. Graphical representations were generated with Pymol (Version 2.5) [55].

## 4. Conclusions

In this work, we reported the structure of MbtI in complex with 5-(3-carboxyphenyl)furan-2-carboxylic acid (**II**), a member of our class of furan-based inhibitors. Although this compound exhibited a lower inhibitory activity, it stabilized the enzyme in a closed configuration, thus leading to a complete picture of the active site region around the ligand. We also described a novel, higher-resolution complex of MbtI with the known inhibitor methyl-AMT, in which the enzyme, conversely, adopted an open conformation. The ensemble of our results supports the hypothesis of a lack of correlation between inhibitor potency and induced closure of the active site lid, while providing an updated frame for future computational efforts and structure-driven optimization studies of furan-based (or similar) compounds.

## Figures and Tables

**Table 1:** Crystallographic data collection and refinement statistics.

Dataset	MbtI-II	MbtI-methyl-AMT
Synchrotron beamline	SOLEIL Proxima 2A	SOLEIL Proxima 2A
Wavelength (Å)	0.9786	0.9801
Space group	P 2_1_	P 2_1_
Unit cell parameters		
*a*, *b*, *c* (Å)	68.09, 84.00, 93.68	95.64, 83.06, 126.26
α, β, γ (°)	90.00, 110.53, 90.00	90.00, 110.66, 90.00
Resolution (Å) ^a^	87.74–1.58 (1.72–1.58)	117.35–1.54 (1.68–1.54)
Unique reflections ^a^	93,645 (4683)	211,264 (10563)
*R*_pim_ ^b^	0.031 (0.454)	0.027 (0.339)
*I*/σ(*I)*	12.7 (1.6)	12.6 (1.6)
Completeness (%) ^c^	94.6 (63.8)	94.7 (62.1)
CC(1/2)	0.998 (0.625)	0.998 (0.752)
Multiplicity	6.9 (6.9)	7.0 (6.2)
**Refinement**		
Resolution (Å)	1.58	1.54
No. reflections	93,600	211,264
*R*_work_/*R*_free_ (%) ^d^	17.9/20.5	18.3/20.8
No. atoms		
Protein	6671	12,855
Ligands/ions	60	127
Solvent	847	1418
Average B-factors		
Protein	29.88	32.95
Ligands/ions	40.82	35.66
Solvent	40.99	41.90
R.m.s. deviations from ideal ^e^		
Bond lengths (Å)	0.015	0.014
Bond angles (°)	1.578	1.546
Validation ^e^		
MolProbity score	0.88	0.98
Clashscore	1.42	2.07
Rotamer outliers (%)	0.72	0.60
Ramachandran plot ^e^		
Favored (%)	98.73	99.17
Allowed (%)	1.15	0.83
Outliers (%)	0.12	0.00
PDB accession code	8QC4	8QN5

^a^ Resolution limits were determined by applying an anisotropic high-resolution cut-off via STARANISO, part of the autoPROC data processing software [47]; values in parentheses refer to the highest resolution shell. ^b^ R_pim_ = Σ_hkl_ [1/(N-1)]^1/2^Σ_i_|I_i_(hkl) − ⟨I⟩(hkl)|/Σ_hkl_ Σ_i_ I_i_(hkl), where N is the multiplicity, I_i_ is the intensity of reflection i, and ⟨I⟩(hkl) is the mean intensity of all symmetry-related reflections. ^c^ Completeness was computed within an ellipsoidal cut-off surface in reciprocal space, as determined by STARANISO. ^d^ R_work_ = Σ||F_o_| − |F_c_||/Σ|F_o_|, where F_o_ and F_c_ are the observed and calculated structure factor amplitudes. Five percent of the reflections were reserved for the calculation of R_free_. ^e^ Values from MOLPROBITY [53].

## Data Availability

Atomic coordinates and structure factors have been deposited in the Protein Data Bank under accession codes 8QC4 (MbtI-**II**) and 8QN5 (MbtI-methyl-AMT).
